# Stroke risk following traumatic brain injury: Systematic review and meta-analysis

**DOI:** 10.1177/17474930211004277

**Published:** 2021-04-04

**Authors:** Grace M Turner, Christel McMullan, Olalekan Lee Aiyegbusi, Danai Bem, Tom Marshall, Melanie Calvert, Jonathan Mant, Antonio Belli

**Affiliations:** 1 Centre for Patient Reported Outcomes Research, College of Medical and Dental Sciences, 1724 University of Birmingham, Birmingham, UK; 2 NIHR Surgical Reconstruction and Microbiology Research Centre, University Hospitals Birmingham NHS Foundation Trust and 1724 University of Birmingham, Birmingham, UK; 3 Institute of Applied Health Research, 1724 University of Birmingham, Birmingham, UK; 4 NIHR Birmingham Biomedical Research Centre, University Hospitals Birmingham NHS Foundation Trust and 1724 University of Birmingham, Birmingham, UK; 5 Birmingham Health Partners Centre for Regulatory Science and Innovation, 1724 University of Birmingham, Birmingham, UK; 6 NIHR Applied Research Collaboration (ARC) West Midlands, 1724 University of Birmingham, Birmingham, UK; 7 Primary Care Unit, Department of Public Health and Primary Care, 2152 University of Cambridge, Strangeways Research Laboratory, Cambridge, UK

**Keywords:** Traumatic brain injury, traumatic brain injury, stroke, risk, meta-analysis, systematic review

## Abstract

**Background:**

Traumatic brain injury is a global health problem; worldwide, >60 million people experience a traumatic brain injury each year and incidence is rising. Traumatic brain injury has been proposed as an independent risk factor for stroke.

**Aims:**

To investigate the association between traumatic brain injury and stroke risk.

**Summary of review:**

We undertook a systematic review of MEDLINE, EMBASE, CINAHL, and The Cochrane Library from inception to 4 December 2020. We used random-effects meta-analysis to pool hazard ratios for studies which reported stroke risk post-traumatic brain injury compared to controls. Searches identified 10,501 records; 58 full texts were assessed for eligibility and 18 met the inclusion criteria. The review included a large sample size of 2,606,379 participants from four countries. Six studies included a non-traumatic brain injury control group, all found traumatic brain injury patients had significantly increased risk of stroke compared to controls (pooled hazard ratio 1.86; 95% confidence interval 1.46–2.37). Findings suggest stroke risk may be highest in the first four months post-traumatic brain injury, but remains significant up to five years post-traumatic brain injury. Traumatic brain injury appears to be associated with increased stroke risk regardless of severity or subtype of traumatic brain injury. There was some evidence to suggest an association between reduced stroke risk post-traumatic brain injury and Vitamin K antagonists and statins, but increased stroke risk with certain classes of antidepressants.

**Conclusion:**

Traumatic brain injury is an independent risk factor for stroke, regardless of traumatic brain injury severity or type. Post-traumatic brain injury review and management of risk factors for stroke may be warranted.

## Introduction

Traumatic brain injury (TBI) is a global health problem; worldwide, more than 60 million people experience a TBI each year and incidence is rising.^1^ Increased incidence of TBI has been attributed to increased falls in elderly populations, armed conflict, and sports-related injury in high-income countries, and increased road traffic accidents in low-/middle-income countries.^2,3^

Advances in critical care, imaging, and the reorganization of trauma health systems have led to a reduction in TBI-related mortality.^4^ However, increased survival rates results in more people living with the long-term effects of TBI. These long-term effects are wide-ranging, including physical, psychological, and cognitive disabilities, and can cause huge burden at individual, family, and societal levels.^5^ Long-term impacts are not exclusive to severe TBI and are often experienced by patients with mild and moderate TBI.^6^

TBI has also been associated with long-term risk of neurological diseases, including dementia, Parkinson’s disease, Alzheimer’s disease, and epilepsy.^7–10^ TBI has been proposed as an independent risk factor for stroke;^11^ however, to our knowledge, no systematic reviews have explored stroke risk post-TBI. Stroke is a leading cause of death and disability worldwide, but stroke prevention medication and lifestyle change can reduce stroke risk.^12^ Therefore, understanding the association between TBI and stroke is important to help inform healthcare for TBI patients. Characterizing stroke risk post-TBI is particularly important given the changing epidemiology of TBI in high-income countries among the elderly and the fact that older age is an independent risk factor for stroke. The aim of this review was to investigate the association between TBI and risk of stroke.

## Material and methods

The review protocol was registered on PROSPERO prior to conducting literature searches (CRD42019121149).^13^ The review methodology and reporting is in accordance with the preferred reporting items for systematic reviews and meta-analyses (PRISMA) guidance.^14^

### Eligibility criteria

Studies were eligible if they included adult participants (≥18 years) who had experienced a TBI (any severity) and reported frequency, incidence, or risk estimates of stroke and/or transient ischemic attack (TIA) at any time point post-TBI. Studies of mixed populations were eligible if it was possible to extract data of TBI participants.

Eligible study designs were controlled, cohort, cross-sectional, and case control studies. Only peer-reviewed, full text publications or theses were included. To avoid language bias, non-English papers were eligible.

### Information sources and search strategy

Searches were performed with no publication date restrictions in the following bibliographic databases: MEDLINE, EMBASE, CINAHL, and The Cochrane Library. Grey literature was searched from the following sources: Grey Matters by CADTH, OpenGrey, and Epistemonikos. Reference lists of relevant studies were also reviewed. The initial search was performed from inception to 14 December 2018, followed by an updated search from 2018 to 4 December 2020.

A combination of text words and index terms related to the condition (TBI) and the outcome (stroke/TIA) were utilized (Supplementary Figure 1).

### Study selection, data extraction, and critical appraisal

Literature search results were exported to EndNote V.X8.0 (Thomson Reuters, New York) and duplicates removed. Titles and abstracts of search results were screened and full texts obtained for potentially eligible studies. A standardized, pre-determined eligibility criteria checklist was used to select eligible studies (Supplementary Table 1). Data were extracted on study design, population, outcomes, and findings using a standardized, piloted data extraction form (Supplementary Table 2). An adapted version of the reporting of studies conducted using observational routinely collected data checklist was used to assess quality of included studies.^15^

All study selection, data extraction, and quality assessment processes were conducted independently and in duplicate by three authors (GMT, CM, and OLA); discrepancies were resolved by an additional reviewer (AB).

### Data synthesis and statistical analysis

A random effects meta-analysis pooled hazard ratios (HRs) for studies which reported stroke risk post-TBI compared to non-TBI controls. The meta-analysis was performed using Review Manager Version 5.3. (Copenhagen: The Nordic Cochrane Centre, The Cochrane Collaboration, 2014). Studies without a non-TBI comparator were narratively summarized and tables were created to facilitate comparisons. A narrative subgroup analysis was conducted for studies which reported time to stroke onset post-TBI; severity or subtype of TBI; and stroke type (ischemic and hemorrhagic).

### Data availability

Data are available on request.

## Results

**Figure 1.**
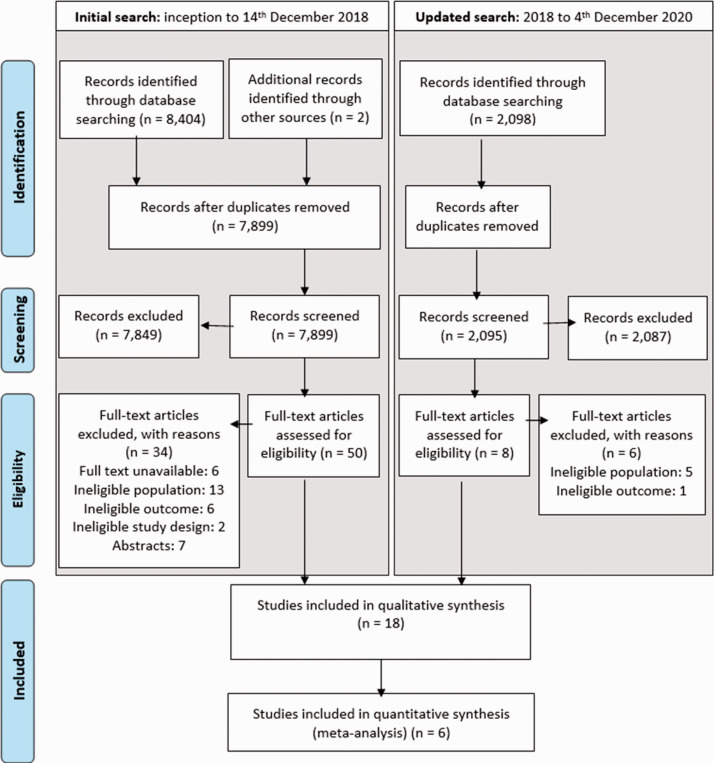
PRISMA flow chart.

Searches identified 10,501 records, of which 58 full texts were assessed for eligibility and 18 met the inclusion criteria (Figure 1).

### Study characteristics

The 18 studies included 2,606,379 participants and were from the United States,^16–24^ Taiwan,^25–31^ Croatia,^32^ and Denmark.^33^ All were retrospective cohort studies and eight different data sources were used (Table 1), most commonly the Taiwan National Health Insurance Research Database^16–18,20,21,31^ and United States Medicare administrative claims data.^24–30^ The eligibility criteria and definitions of TBI and stroke for included studies are summarized in Supplementary Table 3.

**Table 1.** Summary of the country, study period, data source, study arms, age, sex, and TBI severity for included studies (n = 18 studies).

Reference	Country (study period)	Data source	Study arms (sample size)	Age (mean (SD), median (IQR))	Sex (males: n, %)	TBI severity
Stroke risk post-TBI compared to non-TBI controls						
Burke e tal.^19^	USA (2005–2009)	State Inpatient Databases; State Emergency Department Databases; Healthcare Cost and Utilization Project; and Agency for Healthcare Research and Quality	Exposed: TBI patients (n = 436,630) Unexposed: non-TBI trauma patients (n = 736,723)	TBI: 49.2 (22.4) Control: 50.3 (20.1)	TBI: 232,332 (53.2) Control: 373,513 (50.7)	Abbreviated injury scale: mean (SD): TBI: 4.6 (10.1) Control: 4.1 (3.6)
Chen et al.^26^	Taiwan (2001–2003)	Taiwan’s National Health Insurance Research Database: Longitudinal Health Insurance Database 2000	Exposed: TBI patients (n = 23,199) Unexposed: patients without TBI (n = 69,597)	41.6 (18.4)	TBI: 12,431 (53.6) Control: 37,293 (53.6)	NR
Eric Nyam et al.^31^	Taiwan (2000–2012)	Taiwan’s National Health Insurance Research Database: Longitudinal Health Insurance Database 2000	Exposed: TBI patients (n = 16,211) Unexposed: patients without TBI (n = 32,422)	NR	TBI: 9,829 (60.6) Control: 19,676 (60.7)	NR
Lee et al.^27^	Taiwan (2007–2010)	Taiwan’s National Health Insurance Research Database	Exposed: mild TBI patients (n = 24,905) Unexposed: patients without TBI (n = 719,811)	TBI: 46.1 (20.1) Control: 43.5 (16.3)	TBI: 11,814 (47.4) Control: 348,981 (48.5)	Mild
Liao et al.^28^	Taiwan (2000–2004)	Taiwan’s National Health Insurance Research Database	Exposed: TBI patients (n = 30,165) Unexposed: patients without TBI (n = 120,660)	TBI: 44.5 (17.8) Control: 43.9 (17.3)	TBI: 15,202 (50.4) Control: 60,808 (50.4)	Mild: 10,623 Skull fracture: 1281 Severe: 18,261
Liu et al.^29^	Taiwan (1998–2005)	Taiwan’s National Health Insurance Research Database	Exposed: patients with concussion (n = 13,652) Unexposed: patients without concussion (n = 13,652)	Concussion: 56.3 (12.1) Control: 56.2 (12.0)	Concussion: 6267 (45.9) Control: 6279 (46.0)	Mild: concussion
Association of stroke risk post-TBI with specific exposures						
Albrecht et al.^18^	USA (2006–2010)	Administrative claims data from USA Medicare beneficiaries	Exposed: antidepressant use after TBI (n = 15,733) Unexposed: no antidepressant use after TBI (n = 15,153)	79.7 (7.7)	9852 (32)	NR
Khokhar et al.^20^	USA (2006–2010)	Administrative claims data from USA Medicare beneficiaries	Exposed: antidepressant used at least once after TBI (n = 20,859) Unexposed: No antidepressant use after TBI (n = 43,355)	82.8 (8.0)	25,881 (40.2)	NR
Khokhar et al.^21^	USA (2006–2010)	Administrative claims data from USA Medicare beneficiaries	Exposed: statin use after TBI (n = 50,173) Unexposed: no statins used after TBI (n = 50,342)	81.0 (8.1)	34,965 (34.8)	NR
Albrecht et al.^16^	USA (2006–2009)	Administrative claims data from USA Medicare beneficiaries	Exposed: warfarin use after TBI (n = 5811) Unexposed: no warfarin use after TBI (n = 4,971)	81.3 (7.3)	3850 (36)	NR
Staerk et al.^33^	Denmark (2005–2016)	Danish national patient registry; Danish national prescription registry; Danish civil registration system	Exposed: VKAs/NOACs after TBI in AF patients (n = 979) Unexposed: no VKAs/NOACs after TBI in AF patients (n = 421)	79 (72, 85)	1204 (60.4)	NR
Morris et al.^23^	USA (California: 2005–2010; NY: 2006–2013; Florida: 2005–2013)	Inpatient discharges: data from California Office of Statewide Health Planning and Development, NY Statewide Planning and Research Cooperative System, and Florida Agency for Health Care Administration	Exposed: TBI patients with tSAH (n = 20,454) Unexposed: TBI patients without tSAH (n = 20,454)	48.9 (21.6)	TBI with tSAH: 14,826 (72.5) TBI without tSAH: 14,950 (73.0)	NR
Shih et al.^30^	Taiwan (2000–2008)	Taiwan’s National Health Insurance Research Database	Exposed: TBI patients receiving acupuncture (n = 7409) Unexposed: TBI patients without acupuncture (n = 29,636)	Acupuncture: 42.5 (16.9) No acupuncture: 42.6 (17.1)	TBI with acupuncture: 3895 (52.6) TBI without acupuncture: 15,580 (52.6)	Mild: 16,085 Moderate: 7225 Severe: 13,735
Ao et al.^25^	Taiwan (1999–2013)	Taiwan’s National Health Insurance Research Database: Longitudinal Health Insurance Database 2000	Exposed: TBI patients with insomnia (n = 1174) Unexposed: non-TBI patients with insomnia (n = 5870)	51.6 (17.3)	TBI 582 (49.6) Control: 2910 (49.6)	NR
Frequency of stroke post-TBI without a comparator						
Kowalski et al.^22^	USA (2007–2015)	Traumatic Brain Injury Model Systems National Database	TBI patients (no comparator) (n = 6488)	42 (range 16–99)	4725 (73)	Moderate to severe
Belavic et al.^32^	Croatia (2008–2013)	Database of brain injury patients hospitalized in the general ICU at Karlovac General Hospital	TBI patients (no comparator) (n = 306)	56 (range 18–93)	NR	NR
Stroke incidence pre and post-TBI						
Albrecht et al.^17^	USA (2006–2009)	Administrative claims data from USA Medicare beneficiaries	Pre- and post-TBI (n = 16,936)	81.0 (7.9)	6379 (38)	NR
Glass et al.^24^	USA (2006–2009)	Administrative claims data from USA Medicare beneficiaries	TBI patients (no comparator) (n = 52,228)	82.9 (7.9)	18,439 (35.3)	NR

ICU: intensive care units; NOACs: novel oral anticoagulants; NR: not reported; NY: New York; TBI: traumatic brain injury; tSAH: traumatic subarachnoid hemorrhage; USA: United States of America; VKAs: Vitamin K antagonists.

Six studies explored stroke risk post-TBI compared to non-TBI controls (Table 1).^19,26–29,31^ Eight studies investigated the association of stroke risk post-TBI with specific exposures: antidepressants;^18,20^ statins;^21^ oral anticoagulants;^16,33^ traumatic subarachnoid hemorrhage (tSAH);^23^ acupuncture;^30^ and insomnia.^25^ Two studies reported frequency of stroke post-TBI without a comparator^22,32^ and two studies compared stroke incidence pre- and post-TBI.^17,24^

### Risk of stroke post-TBI

#### Stroke risk compared to non-TBI controls

**Figure 2.**
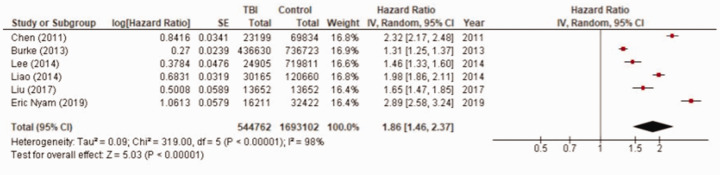
Random effects meta-analysis pooled estimates for stroke risk post-TBI compared to non-TBI controls (n=6 studies; 544,762 TBI patients and 1,692,865 controls).

Of the six studies that included a non-TBI control group, all found TBI patients had statistically significantly increased risk of stroke compared to controls. This association remained when confounding variables were adjusted for (Table 2). All studies matched on or adjusted for age and sex; the list of variables each study adjusted for is detailed in Supplementary Table 4. The pooled adjusted HR was 1.86 (95% confidence interval (CI) 1.46–2.37) (n = 544,762 TBI patients and 1,692,865 controls) (Figure 2).

**Table 2.** Stroke risk compared to non-TBI controls and stroke risk pre-/post-TBI.

Reference	Sample size	Follow-up timeframe	Stroke type	Strokes in follow-up (n, %)	Unadjusted HR (95% CI)	Adjusted* HR (95% CI)
Stroke risk compare to non-TBI controls						
Burke et al.^19^	TBI: 436,630 Control: 736,723	Median years (IQR): 28 months (14–44)	Ischemic	TBI: NR (1.1) Controls: NR (0.9)	NR	1.31 (1.25–1.36)
Chen et al.^26^	TBI: 23,199 Control†: 69,597	3 months, 1 year, 5 years	Ischemic and hemorrhagic	3 months: TBI: 675 (2.91) Controls: 207 (0.30) 1 year: TBI: 968 (4.17) Controls: 669 (0.96) 5 years: TBI: 1901 (8.20) Controls: 2710 (3.89)	3 months: 10.20 (8.71–11.93) 1 year: 4.61 (4.17–5.11) 5 years: 2.34 (2.20–2.50)	3 months: 10.21 (8.71–11.96) 1 year: 4.61 (4.16–5.11) 5 years: 2.32 (2.17–2.47)
Eric Nyam et al.^31^	TBI: 16,211 Control: 32,422§	Median: 5 years, range 0.0027 to 5 years	Ischemic, hemorrhagic and unspecified	TBI: 637 (3.93) Control: 529 (1.63)	NR	All strokes: 2.89 (2.58–3.25) Ischemic: 2.10 (1.81–2.43) Hemorrhagic: 6.02 (4.80–7.55)
Lee et al.^27^	TBI: 24,905 Control: 719,811	Mean years (SD): TBI: 1.94 (1.18) Control: 3.88 (0.55) Person years: TBI 48,371 Control: 2,793,892	Ischemic	TBI: 412 (1.65) Controls: 9242 (1.34)	2.49 (2.25–2.74)	1.46 (1.33–1.62)
Liao et al.^28^	TBI: 30,165 Control‡: 120,660	Max 4 years	Ischemic and hemorrhagic	TBI: 1455 (4.8) Controls: 2903 (2.4)	NR	1.98 (1.86–2.11)
Liu et al.^29^	TBI: 13,652 Control¥: 13,652	Person years: 154,657	Ischemic and hemorrhagic	TBI: 779 Controls: 527	1.48 (1.32–1.66)	1.65 (1.47–1.85)
Stroke risk pre/ post-TBI						
Albrecht et al.^17^	TBI: 16,936	Min 6 months, max 48 months	Ischemic and hemorrhagic	Incidence rate per 1000 Ischemic: Pre-TBI: 70.5 Post-TBI: 84.4 Hemorrhagic: Pre-TBI: 3.7 Post-TBI: 23.9	NR	Rate ratio Ischemic: 1.3 (1.2–1.4) Hemorrhagic: 6.5 (5.3–7.8)
Glass et al.^24^	TBI: 52,228	12 months	Ischemic and hemorrhagic	Annual incidence rate/1000 Ischemic: Pre-TBI: 49.1 (46.5–51.9) Post-TBI: 37.7 (36.0–39.6) Hemorrhagic: Pre-TBI: 13.3 (12.0–14.8) Post-TBI: 16.2 (15.0–17.4)	Rate ratio Ischemic: 0.8 Hemorrhagic: 1.2	*NR*

CI: confidence interval; HR: hazard ratio; IQR: interquartile range; Max: maximum; Min: minimum; n: number; NR: not reported; SD: standard deviation; TBI: traumatic brain injury; %: percentage.

* Variables adjusted for are presented in Supplementary Table 4.

† Matched 1:3 on age, sex, and year of index use of healthcare services.

§Matched 1:2 on age-, sex-, and age-adjusted Charlson Comorbidity Index score.

‡ Matched 1:4 on age and sex.

¥Matched 1:1 on age, sex, and propensity score.

#### Stroke risk pre- and post-traumatic brain injury

Two studies investigated stroke rates pre- and post-TBI.^17,24^ Both studies reported higher incidence rates of ischemic and hemorrhagic stroke post-TBI compared to pre-TBI (Table 2).

#### Stroke risk associated with specified exposures

Three studies explored stroke risk associated with exposure to stroke prevention medication post-TBI (Table 3). For patients who had been prescribed Vitamin K antagonists (VKAs) prior to TBI, continuation or resumption of these drugs after TBI was associated with reduced stroke risk post-TBI compared to patients who had VKA prescriptions stopped post-TBI (Albrecht et al.:^16^ relative risk 0.83; 95% CI 0.72–0.96 and Staerk et al.:^33^ HR 0.58; 95% CI 0.37–0.90). However, no association was found for continuation or resumption of novel oral anticoagulants (NOACs) (HR 0.85; 95% CI 0.43–1.68). Prescription of statins post-TBI was associated with reduced risk compared to TBI patients not prescribed statins post-TBI (relative risk 0.86; 95% CI 0.81–0.91).

**Table 3.** Stroke risk post-TBI associated with specific exposures: anticoagulants, statins, antidepressants, insomnia, tSAH, and acupuncture.

References	Exposure	Sample size	Follow-up timeframe	Strokes in follow-up (n, %)	Unadjusted risk estimate (95% CI)	Adjusted risk estimate (95% CI)
Albrecht et al.^16^	Warfarin use post-TBI	Exposed: 5811 Unexposed: 4971	12 months	Events, n* Any stroke: Exposed: 339 Unexposed: 494 Ischemic: Exposed: 293 Unexposed: 453 Hemorrhagic: Exposed: 75 Unexposed: 130	NR	Relative risk Any stroke: 0.83 (0.72–0.96) Ischemic: 0.81 (0.69–0.95) Hemorrhagic: 0.70 (0.52–0.95)
Staerk et al.^33^	VKAs/NOACs use post-TBI	Exposed: 979 Unexposed: 421	3 years	NR	NR	Hazard ratio VKA: 0.58 (0.37–0.90) NOAC: 0.85 (0.43–1.68)
Khokhar et al.^21^	Statin use post-TBI	Exposed: 50,173 Unexposed: 50,342	Up to 54 months	Ischemic: 9420 Hemorrhagic: 3841	Relative risk Any stroke: 1.00 (0.96–1.04) Ischemic: 1.09 (1.04–1.14) Hemorrhagic: 0.81 (0.75–0.87)	Relative risk Any stroke: 0.86 (0.81–0.91) Ischemic: 0.91 (0.85–0.96) Hemorrhagic: 0.75 (0.67–0.83)
Khokhar et al.^20^	Antidepressant use post-TBI	Exposed: 20,859 Unexposed: 43,355	Mean days (SD): Exposed: 719.5 (393.7) Unexposed: 621.5 (426.5)	Ischemic: Exposed: 2413 (11.7) Unexposed: 3121 (7.5) Hemorrhagic: Exposed: 949 (4.6) Unexposed: 1448 (3.4)	Relative risk Any stroke: SSRI 1.27 (1.16–1.39) SNRI 1.62 (1.30–2.02) TCA 1.15 (0.82–1.61) TetCA 1.16 (0.96–1.41) PPA 1.48 (1.18–1.84) Ischemic: SSRI 1.29 (1.17–1.42) SNRI 1.70 (1.34–2.16) TCA 1.14 (0.78–1.67) TetCA 1.23 (0.99–1.52) PPA 1.48 (1.15–1.90) Hemorrhagic: SSRI 1.33 (1.13–1.57) SNRI 1.20 (0.73–1.96) TCA 1.09 (0.57–2.10) TetCA 0.91 (0.60–1.38) PPA 1.44 (0.96–2.18)	Relative risk Any stroke: SSRI 1.07 (0.98–1.18) SNRI 1.37 (1.10–1.72) TCA 1.13 (0.81–1.59) TetCA 0.96 (0.79–1.17) PPA 1.33 (1.07–1.66) Ischemic: SSRI 1.04 (0.94–1.15) SNRI 1.36 (1.06–1.73) TCA 1.11 (0.76–1.63) TetCA 0.95 (0.77–1.18) PPA 1.31 (1.02–1.68) Hemorrhagic: SSRI 1.26 (1.06–1.50) SNRI 1.19 (0.72–1.95) TCA 1.11 (0.58–2.14) TetCA 0.84 (0.55–1.27) PPA 1.30 (0.86–1.97)
Albrecht et al.^18^	Antidepressant use post-TBI	Exposed: 15,733 Unexposed: 15,153	2 years; person-months of antidepressant use: SSRI: 163,212 SNRI: 24,756 TCA: 19,734 Unexposed: 438,781	Ischemic: SSRI: 2,142 (1.31) SNRI: 466 (1.88) TCA: 243 (1.23) Unexposed: 5243 (1.19) Hemorrhagic: SSRI: 431 (0.26) SNRI: 96 (0.39) TCA: 41 (0.21) Unexposed: 1065 (0.24)	Risk ratios Ischemic: SSRI vs. SNRI (ref SNRI): 1 (0.88–1.12) SSRI vs. TCA (ref TCA): 1.21 (1.02–1.44) SNRI vs. TCA (ref TCA): 1.17 (0.99–1.38) Hemorrhagic: SSRI vs. SNRI (ref SNRI): 0.95 (0.73–1.24) SSRI vs. TCA (ref TCA): 1.69 (1.05–2.70) SNRI vs. TCA (ref TCA): 1.60 (1.05–2.46)	Risk ratios Ischemic: SSRI vs. SNRI (ref SNRI): 0.92 (0.73–1.15) SSRI vs. TCA (ref TCA): 0.99 (0.76–1.31) SNRI vs. TCA (ref TCA): 0.98 (0.73–1.33) Hemorrhagic: SSRI vs. SNRI (ref SNRI): 1.17 (0.71–1.92) SSRI vs. TCA (ref TCA): 2.47 (1.30–4.70) SNRI vs. TCA (ref TCA): 1.39 (0.59–3.25)
Ao et al.^25^	New onset insomnia post-TBI vs. insomnia without TBI	Exposed: 1174 Unexposed: 5870	Mean years (SD): 2.71 (0.69)	TBI and insomnia: 65 (5.54) Insomnia: 144 (2.45)	2.33 (1.74–3.12)	2.28 (1.70–3.06)
Morris et al.^23^	tSAH post-TBI	Exposed: 20,454 Unexposed: 20,454	Mean years (SD) 4.3 (1.8)	Total sample: 531 (1.3) Cumulative stroke rate: Exposed: 1.79% (1.54– 2.08) Unexposed: 2.12% (1.83–2.4)	Hazard ratio 0.77 (0.63–0.94)	NR
Shih et al.^30^	TBI patients receiving acupuncture post-TBI	Exposed: 7409 Unexposed: 29,636	Person-years: Exposed: 33,071 Unexposed: 17,3682	Exposed: 163 (2.2) Unexposed: 1250 (4.2)	NR	Hazard ratio 0.59 (0.50–0.69)

CI: confidence interval; n: number; NOACs: novel oral anticoagulants; NR: not reported; PPA: phenylpiperazine antidepressants SD: standard deviation; SNRI: serotonin norepinephrine reuptake inhibitors; SSRI: selective serotonin reuptake inhibitors; TBI: traumatic brain injury; TCA: tricyclic antidepressants; TetCA: tetracyclic antidepressants; tSAH: traumatic subarachnoid hemorrhage; VKAs: Vitamin K antagonists; %: percentage.

* Note: numbers do not add up as a result of exclusion of events occurring during hospitalization for TBI and multiple simultaneous events.

Two studies investigated antidepressant use in TBI patients aged ≥ 65 years, both used administrative claims data from United States Medicare beneficiaries between 2006 and 2010. Khokhar et al.^20^ reported, compared to no use of antidepressants post-TBI, new use (i.e. not prescribed before TBI) of serotonin norepinephrine reuptake inhibitors (SNRIs) and phenylpiperazine antidepressants (PPAs) were associated with increased risk of ischemic stroke (relative risk 1.36; 95% CI 1.06–1.73 and 1.31; 95% CI 1.02–1.68, respectively). They found new use of selective serotonin reuptake inhibitors (SSRIs) was associated with increased risk of hemorrhagic stroke (relative risk 1.26; 95% CI 1.06–1.50). However, no associations were found for other antidepressant drug classes (Table 3). Albrecht et al.^18^ found, compared to tricyclic antidepressants (TCAs), SSRI use was associated with increased risk of hemorrhagic stroke (risk ratio 2.47; 95% CI 1.30–4.70). However, other antidepressant drug class comparisons were not associated with stroke risk (Table 3).

Exposures reported in the other three studies were insomnia,^25^ acupuncture,^30^ and tSAH.^23^ TBI patients with new onset insomnia (i.e. insomnia occurring post-TBI) had a 2-fold increased stroke risk (HR 2.28; 95% CI 1.70–3.06) compared to insomnia patients without TBI.^25^ Acupuncture treatment post-TBI was associated with decreased stroke risk compared to TBI patients not receiving acupuncture (HR 0.59; 95% CI 0.50–0.69).^30^ TBI patients with tSAH had decreased stroke risk compared to TBI patients without tSAH (HR 0.77; 95% CI 0.63–0.94).

### Time to stroke onset post-TBI

Chen et al.^26^ found stroke risk post-TBI was highest in the first three months of follow-up (HR 10.21; 95% CI 8.71–11.96), but remained significantly higher than non-TBI controls at five years follow-up (HR 2.32; 95% CI 2.17–2.47) (Table 2).

Albrecht et al.^17^ reported stroke incidence rates for TBI patients were highest two and four month’s post-discharge and there was a steep decrease between four and 12 months post-discharge: incidence rates (per 1000) for ischemic stroke at two months 183.5; four months 112.3; six months 84.8; eight months 69.9; 10 months 70.3; 12 months 78.1, and hemorrhagic stroke at two months 98.5; four months 36.1; six months 17.3; eight months 11.7; 10 months 12.8; 12 months 8.2.

Similarly, Albrecht et al.^17^ found stroke rates post-TBI were elevated in the first three months after discharge and leveled out between three and 24 months for ischemic stroke and 12 and 24 months for hemorrhagic stroke (Supplementary Figure 2).

In a subgroup analysis of people who survived longer than 12 months, Lee et al.^27^ found TBI patients’ stroke risk, compared to non-TBI controls, remained statistically significant (HR 1.38; 95% CI, 1.20–1.59).

Four studies reported average time between TBI and onset of stroke: Ao et al.^25^ 0.93 years (mean follow-up 2.71 years; standard deviation (SD) 0.69); Belavic et al.^32^ 8.56 days (mean follow-up 29.13 days; SD 27.16); Lee et al.^27^ 1.12 years (mean follow-up 1.94 years; SD 1.18); Chen et al.^26^ 1.49 years (maximum follow-up five years).

Two studies reported frequency of stroke diagnosed during acute hospitalization for TBI. Kowalski et al.^22^ found 2.5% (159/6488) of moderate to severe TBI patients had an ischemic stroke (median hospital duration 25 days, range 4–125 days). Belavic et al.^32^ found 7.5% (23/306) of TBI patients had a stroke (mean time to stroke after TBI 7 ± 8 days and mean hospital duration 29 ± 27 days).

### Severity and subtype of TBI

Two studies had populations of mild TBI patients, both reported increased stroke risk post-TBI compared to non-TBI controls: Lee et al.^27^ HR 1.46 (95% CI 1.33–1.62) and Liu et al.^29^ HR 1.65 (95% CI 1.47–1.85) (Table 2). In a secondary analysis stratified by trauma severity, Burke et al.^19^ found ischemic stroke risk remained significantly higher than controls regardless of severity (tertile 1 (lowest severity): HR 1.10; 95% CI 1.01–1.20, tertile 2: HR 1.29; 95% CI 1.16–1.43, tertile 3 (highest severity): HR 1.25; 95% CI 1.16–1.35). Similarly, Liao et al.^28^ found increased risk of stroke post-TBI compared to controls for all types of TBI: mild TBI: HR 1.74 (95% CI 1.57–1.93); severe TBI: HR 2.04 (95% CI 1.89–2.20); skull fracture: HR 3.00 (95% CI 2.42–3.71).

In a secondary analysis of trauma subtype, Burke et al.^19^ found increased ischemic stroke risk compared to controls regardless of subtype: skull fracture HR 1.21 (95% CI 1.05–1.41); concussion HR 1.27 (95% CI 1.17–1.37); intracranial bleeding HR 1.21 (95% CI 1.12–1.31); other intracranial injury HR 1.38 (95% CI 1.07–1.76); and unspecified HR 1.33 (95% CI 1.27–1.40). Chen et al.^26^ found stroke risk, compared to controls, was more pronounced for TBI patients with skull fracture at three months (skull fracture: HR 19.98; 95% CI 14.73–27.22 vs. without skull fracture: HR 9.75; 95% CI 8.31–11.45), one year (skull fracture: HR 8.39; 95% CI 7.47–10.89 vs. without skull fracture: HR 4.44; 95% CI 4.00–4.93), and five years (skull fracture: HR 3.54; 95% CI 2.86–4.37 vs. without skull fracture: HR 2.26; 95% CI 2.12–2.42).

### Stroke type

Five studies reported stroke risk post-TBI by stroke type. Eric Nyam et al.^31^ found higher risk of hemorrhagic stroke post-TBI, compared to controls, than ischemic stroke (HR 6.02; 95% CI 4.80–7.55 and HR 2.10; 95% CI 1.81–2.43, respectively) Chen et al.^26^ reported higher risk of stroke post-TBI, compared to controls, for subarachnoid and intra-cerebral hemorrhagic stroke types than ischemic and unspecific strokes (subarachnoid hemorrhage: HR 4.89; 95% CI 3.81–7.19, intra-cerebral hemorrhage: HR 6.33; 95% CI 5.60–7.83, ischemic: HR 1.43; 95% CI 1.31–1.56, unspecified: 2.21; 95% CI 1.99–2.44). Similarly, Albrecht et al.^17^ and Glass et al.^24^ found higher incidence rate ratios for hemorrhagic stroke post-TBI, compared to pre-TBI, than for ischemic stroke (Table 2). In contrast, Liu et al.^29^ found higher cumulative incidence rates post-TBI for ischemic stroke compared to hemorrhagic stroke (8.9%; 95% CI 7.8–10.0 vs. 2.7%; 95% CI 2.1–3.3, respectively) and similar adjusted HRs for stroke risk post-TBI, compared to controls, for both stroke subtypes (ischemic: HR 1.62; 95% CI 1.43–1.84 vs. hemorrhagic: HR 1.73; 95% CI 1.36–2.20).

### Study quality

Critical appraisal of included studies (Supplementary Table 5) identified that the study design – including setting, recruitment, and follow-up dates – and eligibility criteria were well described by all included studies. All studies, except one, clearly reported the clinical codes used to identify exposures and outcomes; however, only three studies referenced validation studies for these clinical codes and nine studies did not provide a complete list of clinical codes for confounders and other variables. Statistical methods were described well by most studies; however, none of the studies explained how missing data were addressed. Furthermore, none of the studies clearly described data cleaning methods and most studies (n = 10) did not report population selection based on data quality, data availability, and linkage.

## Discussion

This systematic review is the first to explore the association between TBI and stroke risk. The meta-analysis found TBI patients have 86% increased risk of stroke compared to non-TBI controls (HR 1.86; 95% CI 1.46–2.37). Stroke risk may be highest in the first four months post-TBI, but remains significant five years post-TBI. TBI is associated with increased stroke risk regardless of TBI severity or subtype. Furthermore, TBI is associated with increased risk of both ischemic and hemorrhagic stroke. VKAs, statins, and acupuncture use is associated with reduced stroke risk post-TBI; however, no association was found for NOAC use. Some classes of antidepressants – SNRIs, PPAs, and SSRIs – are associated with increased stroke risk post-TBI.

Our findings suggest TBI is an independent risk factor for stroke regardless of TBI severity or subtype, even if it is mild and patients experience a good recovery. This is particularly noteworthy because 70–90% of TBIs are mild.^34^ Stroke is the second leading cause of death and third leading cause of disability worldwide; however, urgent treatment can prevent stroke-related death and long-term disability.^35,36^ Therefore, it may be beneficial to inform TBI patients of their potential increased stroke risk and educate them to recognize and respond urgently to stroke symptoms.

Primary stroke prevention is important to reduce stroke incidence and subsequent stroke-related death and disability; therefore, clinicians should review patients’ stroke risk post-TBI and consider administering stroke prevention medication and lifestyle advice. Our review found some evidence to suggest an association between reduced stroke risk post-TBI and the stroke prevention drugs VKAs and statins. Furthermore, Khokhar et al.^21^ found statin use post-TBI was also associated with decreased mortality, depression, Alzheimer’s disease, and related dementias. However, stroke prevention drugs are often stopped after an individual has experienced a TBI; Albrecht et al.^16^ found 55% of patients who had been prescribed warfarin prior to TBI were not prescribed warfarin post-TBI,^16^ and Orlando^37^ found a statin discontinuation rate of 38% post-TBI for patients who took statins prior to TBI.^37^ Other research has found older age and risk of falls are common barriers to clinicians’ prescribing stroke prevention drugs.^38^ This is particularly relevant given the shift in TBI epidemiology in high-income countries to falls in the elderly.^3^ Future research is required to investigate the effectiveness of stroke prevention medication post-TBI and related adverse events to help inform clinicians’ prescribing and facilitate shared decision making. Future research should also explore clinical and demographic characteristics associated with increased stroke risk post-TBI to identify individuals most at risk of stroke post-TBI and help clinicians’ tailor preventative healthcare.

Stroke risk may be highest in the first four months post-TBI; therefore, this time period is critical to educate patients about stroke risk and symptoms, and to administer stroke prevention medication and lifestyle advice. Importantly, stroke prevention medication, particularly VKAs and statins, should be re-started for patients who were prescribed these drugs prior to TBI. However, sequelae from TBI, such as physical and cognitive disability, may impair patients’ ability to engage with lifestyle change, adhere to medication, or recognize and respond to stroke symptoms. Therefore, rehabilitation post-TBI, including the role of carers and family members, should provide ongoing support for stroke prevention. Future research is required to further understand the temporal nature of stroke risk post-TBI.

Our review found some evidence to suggest an association between some classes of anti-depressants and increased stroke risk post-TBI. This potential association is important given that many people experience anxiety and depression post-TBI,^5^ which are often treated with antidepressants. Khokhar et al.^20^ found that a third of TBI patients were prescribed new use of antidepressants. A recent systematic review found antidepressant use is associated with increase stroke risk (risk ratio 1.41; 95% CI 1.13–1.69).^39^ However, a study published since that review suggested antidepressants strongly inhibiting serotonin reuptake may be associated with a small decrease in the rate of ischemic stroke.^40^ Further research is required into the safety of antidepressants specifically for TBI patients.

The mechanism for increased stroke risk post-TBI is unknown and is an area for future research; however, a number of hypotheses have been suggested. Damage to the cerebrovascular system caused by TBI could cause stroke through clot formation, damaged arteries, dissection, or increased intracranial pressure and blood pressure.^26^ This could be identified by routine screening for damage to the cerebral vasculature post-TBI and is an area for future research. Alternatively, increased stroke risk may be due to lifestyle changes post-TBI, such as a more sedentary lifestyle due to physical, cognitive, or psychological long-term effects.^28^ Finally, TBI patients may be more likely to be prescribed antipsychotic and antidepressant drugs for TBI-related psychiatric and psychological disorders. These drugs have been associated with increased stroke risk.^18,21,41^

The review is methodologically robust; all screening, data extraction, and quality assessment processes were completed in duplicate. Non-English language papers were eligible to reduce language bias, although none were eligible. However, only peer-reviewed journal articles or theses were eligible which may introduce publication bias. The review included a large sample size of 2,606,379 participants from four countries. However, only six out of the 18 studies compared stroke risk to non-TBI controls and studies were heterogeneous in terms of study population and duration of follow-up. Furthermore, all included studies were retrospective cohort studies which used data collected for routine clinical practice. Potential sources of bias from use of routine data for observational research include misclassification bias, missing data, unmeasured confounding, and changes in coding practices or database eligibility criteria.^15^ In addition, information on stroke type, TBI classification, and TBI severity were missing from the majority of studies. The association of stroke risk with treatment (in both directions) may be confounded by indication bias. Therefore, the review findings should be interpreted cautiously. The included studies were generally good quality in terms of study design and reporting; however, none of the studies clearly reported data cleaning processes and there was a lack of reporting of population selection based on data quality, availability, and linkage. This information is important because choice of data cleaning methods and population selection can affect study findings and reproducibility. Finally, most of the studies included administrative databases, which implies that the TBI definition might be inconsistent. However, in practice, these databases provide large sample sizes, thereby increasing reliability and validity.

Our findings suggest TBI is an independent risk factor for stroke, regardless of TBI severity or subtype. There is some evidence to suggest stroke risk may be highest in the first four months post-TBI; however, it remains increased up to five years post-TBI. VKAs and statins may reduce stroke risk post-TBI; however, they are frequently stopped post-TBI. Post-TBI review and management of risk factors for stroke may be warranted to mitigate the excess risk of stroke associated with TBI. Future research should investigate which subgroups are most at risk of stroke post-TBI; the temporal nature of stroke risk post-TBI; the effectiveness of stroke prevention medication post-TBI; the safety of antidepressants post-TBI; and the mechanism of stroke risk post-TBI.

## References

[1] Dewan MC Rattani A Gupta S , et al. Estimating the global incidence of traumatic brain injury. J Neurosurg 2018, pp. 1–18. 10.3171/2017.10.JNS17352 29701556

[2] Maas AI Stocchetti N Bullock R . Moderate and severe traumatic brain injury in adults. Lancet Neurol 2008; 7: 728–741. 18635021 10.1016/S1474-4422(08)70164-9

[3] Brazinova A Rehorcikova V Taylor MS , et al. Epidemiology of traumatic brain injury in Europe: a living systematic review. J Neurotrauma 2018. doi: 10.1089/neu.2015.4126. 10.1089/neu.2015.4126 PMC8082737 26537996

[4] Ghajar J . Traumatic brain injury. Lancet (London, England) 2000; 356: 923–929. 10.1016/S0140-6736(00)02689-1 11036909

[5] Levack WM Kayes NM Fadyl JK . Experience of recovery and outcome following traumatic brain injury: a metasynthesis of qualitative research. Disabil Rehabil 2010; 32: 986–999. 20450406 10.3109/09638281003775394

[6] Millis SR Rosenthal M Novack TA , et al. Long-term neuropsychological outcome after traumatic brain injury. J Head Trauma Rehabil 2001; 16: 343–355. 11461657 10.1097/00001199-200108000-00005

[7] Julien J Joubert S Ferland MC , et al. Association of traumatic brain injury and Alzheimer disease onset: a systematic review. Ann Phys Rehabil Med 2017; 60: 347–356. 28506441 10.1016/j.rehab.2017.03.009

[8] Li Y Li Y Li X , et al. Head injury as a risk factor for dementia and Alzheimer’s disease: a systematic review and meta-analysis of 32 observational studies. PLoS One 2017; 12: e0169650. 28068405 10.1371/journal.pone.0169650 PMC5221805

[9] Marras C Hincapié CA Kristman VL , et al. Systematic review of the risk of Parkinson's disease after mild traumatic brain injury: results of the international collaboration on mild traumatic brain injury prognosis. Arch Phys Med Rehabil 2014; 95: S238–S244. 24581909 10.1016/j.apmr.2013.08.298

[10] Perry DC Sturm VE Peterson MJ , et al. Association of traumatic brain injury with subsequent neurological and psychiatric disease: a meta-analysis. J Neurosurg 2016; 124: 511. 26315003 10.3171/2015.2.JNS14503 PMC4751029

[11] Maas AIR Menon DK Adelson PD , et al. Traumatic brain injury: integrated approaches to improve prevention, clinical care, and research. Lancet Neurol 2017; 16: 987–1048. 29122524 10.1016/S1474-4422(17)30371-X

[12] Johnson CO Nguyen M Roth GA , et al. Global, regional, and national burden of stroke, 1990-2016: a systematic analysis for the Global Burden of Disease Study 2016. Lancet Neurol 2019; 18: 439–458. 30871944 10.1016/S1474-4422(19)30034-1 PMC6494974

[13] Turner G, Calvert M, Mant J, Belli T and Bem D. Risk of stroke after traumatic brain injury (TBI): a systematic review. PROSPERO 2019 CRD42019121149, 2018, www.crd.york.ac.uk/PROSPERO/display_record.php?ID=CRD42019121149 (accessed 17 March 2021).

[14] Moher D, Liberati A, Tetzlaff J, and Altman DG. Preferred reporting items for systematic reviews and meta-analyses (PRISMA), http://prisma-statement.org/PRISMAStatement/Checklist.aspx (accessed 17 March 21).

[15] Maas AIR Menon DK Adelson PD , et al. Traumatic brain injury: integrated approaches to improve prevention, clinical care, and research. Lancet Neurol 2017; 16: 987–1048. 29122524 10.1016/S1474-4422(17)30371-X

[16] Albrecht JS Liu X Baumgarten M , et al. Benefits and risks of anticoagulation resumption following traumatic brain injury. JAMA Internal Med 2014; 174: 1244–1251. 24915005 10.1001/jamainternmed.2014.2534 PMC4527047

[17] Albrecht JS Liu X Smith GS , et al. Stroke incidence following traumatic brain injury in older adults. J Head Trauma Rehabil 2015; 30: E62–E67. 24816156 10.1097/HTR.0000000000000035 PMC4524572

[18] Albrecht JS Rao V Perfetto EM Daniel Mullins C . Safety of antidepressant classes used following traumatic brain injury among medicare beneficiaries: a retrospective cohort study. Drugs Aging 2018; 35: 763–772. 30047070 10.1007/s40266-018-0570-2 PMC12744690

[19] Burke JF Stulc JL Skolarus LE Sears ED Zahuranec DB Morgenstern LB . Traumatic brain injury may be an independent risk factor for stroke. Neurology 2013; 81: 33–39. 23803315 10.1212/WNL.0b013e318297eecf PMC3770205

[20] Khokhar B Simoni-Wastila L Albrecht JS . Risk of stroke among older medicare antidepressant users with traumatic brain injury. J Head Trauma Rehabil 2017; 32: E42–E49. 27022963 10.1097/HTR.0000000000000231 PMC5040609

[21] Khokhar B Simoni-Wastila L Slejko JF Perfetto E Zhan M Smith GS . Mortality and associated morbidities following traumatic brain injury in older medicare statin users. J Head Trauma Rehabil 2018; 33: E68–E76. 29385012 10.1097/HTR.0000000000000369 PMC6066463

[22] Kowalski RG Haarbauer-Krupa JK Bell JM , et al. Acute ischemic stroke after moderate to severe traumatic brain injury: incidence and impact on outcome. Stroke 2017; 48: 1802–1809. 28611087 10.1161/STROKEAHA.117.017327 PMC6025795

[23] Morris NA Cool J Merkler AE Kamel H . Subarachnoid hemorrhage and long-term stroke risk after traumatic brain injury. Neurohospitalist 2017; 7: 122–126. 28634501 10.1177/1941874416675796 PMC5467817

[24] Glass NE Vadlamani A Hwang F , et al. Bleeding and thromboembolism after traumatic brain injury in the elderly: a real conundrum. J Surg Res 2019; 235: 615–620. 30691850 10.1016/j.jss.2018.10.021 PMC12726834

[25] Ao K-H Ho C-H Wang C-C Wang J-J Chio C-C Kuo J-R . The increased risk of stroke in early insomnia following traumatic brain injury: a population-based cohort study. Sleep Med 2017; 37: 187–192. 28899533 10.1016/j.sleep.2017.02.010

[26] Chen Y-H Kang J-H Lin H-C . Patients with traumatic brain injury: population-based study suggests increased risk of stroke. Stroke 2011; 42: 2733–2739. 21799162 10.1161/STROKEAHA.111.620112

[27] Lee YK Lee CW Huang MY Hsu CY Su YC . Increased risk of ischemic stroke in patients with mild traumatic brain injury: a nationwide cohort study. Scand J Trauma Resuscitation Emerg Med 2014; 22: 66. 10.1186/s13049-014-0066-y PMC4239396 25406859

[28] Liao C-C Chou Y-C Yeh C-C Hu C-J Chiu W-T Chen T-L . Stroke risk and outcomes in patients with traumatic brain injury: 2 nationwide studies. Mayo Clinic Proc 2014; 89: 163–172. 10.1016/j.mayocp.2013.09.019 24485130

[29] Liu S-W Huang L-C Chung W-F , et al. Increased risk of stroke in patients of concussion: a nationwide cohort study. Int J Environ Res Public Health 2017; 14: 230. doi:10.3390/ijerph14030230. 10.3390/ijerph14030230 PMC5369066 28245607

[30] Shih C-C Hsu Y-T Wang H-H , et al. Decreased risk of stroke in patients with traumatic brain injury receiving acupuncture treatment: a population-based retrospective cohort study. PLoS One 2014; 9: e89208. 24586597 10.1371/journal.pone.0089208 PMC3929662

[31] Eric Nyam TT Ho CH Chio CC , et al. Traumatic brain injury increases the risk of major adverse cardiovascular and cerebrovascular events: a 13-year, population-based study. World Neurosurg 2019; 122: e740–e753. 30391613 10.1016/j.wneu.2018.10.130

[32] Belavic M Jancic E Miskovic P Brozovic-Krijan A Bakota B Zunic J . Secondary stroke in patients with polytrauma and traumatic brain injury treated in an Intensive Care Unit, Karlovac General Hospital, Croatia. Injury 2015; 46: S31–S35. 10.1016/j.injury.2015.10.057 26620118

[33] Staerk L Fosbol EL Lamberts M , et al. Resumption of oral anticoagulation following traumatic injury and risk of stroke and bleeding in patients with atrial fibrillation: a nationwide cohort study. Eur Heart J 2018; 39: 1698–1705. 29165556 10.1093/eurheartj/ehx598

[34] Feigin VL Theadom A Barker-Collo S , et al. Incidence of traumatic brain injury in New Zealand: a population-based study. Lancet Neurol 2013; 12: 53–64. 23177532 10.1016/S1474-4422(12)70262-4

[35] Lozano R Naghavi M Foreman K , et al. Global and regional mortality from 235 causes of death for 20 age groups in 1990 and 2010: a systematic analysis for the Global Burden of Disease Study 2010. Lancet (London, England) 2012; 380: 2095–2128. 10.1016/S0140-6736(12)61728-0 PMC10790329 23245604

[36] Murray CJ Vos T Lozano R , et al. Disability-adjusted life years (DALYs) for 291 diseases and injuries in 21 regions, 1990-2010: a systematic analysis for the Global Burden of Disease Study 2010. Lancet (London, England) 2012; 380: 2197–2223. 10.1016/S0140-6736(12)61689-4 23245608

[37] Orlando A Bar-Or D Salottolo K , et al. Unintentional discontinuation of statins may increase mortality after traumatic brain injury in elderly patients: a preliminary observation. J Clin Med Res 2013; 5: 168–173. 23671542 10.4021/jocmr1333w PMC3651067

[38] Pugh D Pugh J Mead GE . Attitudes of physicians regarding anticoagulation for atrial fibrillation: a systematic review. Age Ageing 2011; 40: 675–683. 21821732 10.1093/ageing/afr097

[39] Trajkova S d'Errico A Soffietti R Sacerdote C Ricceri F . Use of antidepressants and risk of incident stroke: a systematic review and meta-analysis. Neuroepidemiology 2019; 53: 142–151. 31216542 10.1159/000500686

[40] Douros A Dell'Aniello S Dehghan G Boivin J-F Renoux C . Degree of serotonin reuptake inhibition of antidepressants and ischemic risk. A cohort study. Neurology 2019; 93: e1010–e1020. 31391245 10.1212/WNL.0000000000008060 PMC6745737

[41] Gill SS Rochon PA Herrmann N , et al. Atypical antipsychotic drugs and risk of ischaemic stroke: population based retrospective cohort study. BMJ 2005; 330: 445. 15668211 10.1136/bmj.38330.470486.8F PMC549652

